# Supplementary material for: Depth-dependent anisotropy in the Earth’s inner core linked to chemical stratification
Source: Nat Commun. 2025 Dec 8;16:10986. doi: 10.1038/s41467-025-67067-y (PMC12689643; doi:10.1038/s41467-025-67067-y)
Supplement: Supplementary file 1 — Supplementary Information [file 41467_2025_67067_MOESM1_ESM.pdf]

Depth-dependent anisotropy in the Earth's inner core linked to chemical stratification

Efim Kolesnikov[1*], Xiang Li[1], Susanne Müller[1], Arno Rohrbach[1], Stephan Klemme[1], Jasper Berndt[1], Hanns-Peter Liermann[2] and Carmen Sanchez-Valle[1], Ilya Kupenko[1,3]

[1]*Institute for Mineralogy, University of Münster, Münster, Germany*
[2]*Deutsches Elektronen-Synchrotron DESY, Hamburg, Germany*
[3]*ESRF, The European Synchrotron, 71 Avenue des Martyrs, CS40220, 38043 Grenoble Cedex 9, France*

[*]e-mail: ekolesni@uni-muenster.de

Supplementary material

Supplementary Table 1. Pressure (P) evolution of the individual lattice strain parameters $Q(hkl)$ and averaged lattice strain parameter $\langle Q(hkl)\rangle$ of hcp-Fe-2Si-0.4C at 300 K. $\langle Q(hkl)\rangle$ here is obtained over the available planes, i.e. (100), (002) and (103).

P, GPa	ΔP, GPa	$Q(100)$	$\Delta Q(100)$	$Q(002)$	$\Delta Q(002)$	$Q(103)$	$\Delta Q(103)$	$\langle Q(hkl)\rangle$	$\Delta\langle Q(hkl)\rangle$
18	3	0.007	0.0003	0.0092	0.0002	0.0050	0.0004	0.0071	0.0012
41	4	0.0100	0.0005	0.0052	0.0006	0.0060	0.0003	0.0071	0.0015
49.4	0.5	0.0055	0.0006	0.0090	0.0005	0.0070	0.0003	0.0072	0.0011
62	6	0.007	0.001	0.0070	0.0007	0.0074	0.0003	0.0071	0.0004
80	8	0.009	0.001	0.0084	0.0008	0.0080	0.0002	0.0085	0.0005
87	9	0.009	0.001	0.0076	0.0005	0.0055	0.0002	0.0074	0.0011
91	9	0.0100	0.0011	0.0110	0.0011	0.0083	0.0003	0.0098	0.0009
98	10	0.0102	0.0008	0.009	0.001	0.0057	0.0004	0.0083	0.0014
98	10	0.0105	0.0004	0.0070	0.0006	0.0085	0.0005	0.0087	0.0011
105	11	0.0134	0.0014	0.0085	0.0014	0.0064	0.0006	0.0094	0.0022

107	11	0.011	0.001	0.0078	0.0011	0.0068	0.0003	0.0085	0.0014
108	11	0.0107	0.0016	0.0071	0.0006	0.0060	0.0002	0.0079	0.0015
115	12	0.0104	0.0015	0.0061	0.0006	0.0060	0.0002	0.0075	0.0015
128	13	0.009	0.003	0.0065	0.0007	0.0090	0.0006	0.0082	0.0013

Supplementary Table 2. Pressure (P) evolution of the individual lattice strain parameters $Q(hkl)$ and averaged lattice strain parameter $\langle Q(hkl)\rangle$ of hcp-Fe-2Si-0.4C at 1100 K. $\langle Q(hkl)\rangle$ here is obtained over all the available planes, i.e. (100), (002), (101), (102), (110) and (103).

P, GPa	ΔP, GPa	$Q(100)$	$\Delta Q(100)$	$Q(002)$	$\Delta Q(002)$	$Q(101)$	$\Delta Q(101)$	$Q(102)$	$\Delta Q(102)$	$Q(110)$	$\Delta Q(110)$	$Q(103)$	$\Delta Q(103)$	$\langle Q(hkl)\rangle$	$\Delta\langle Q(hkl)\rangle$
36.33	0.05	0.0073	0.0003	0.0082	0.0001	0.0076	0.0001	0.0018	0.0003	0.0031	0.0004	0.0053	0.0003	0.0056	0.0011
37.28	0.05	0.0080	0.0003	0.0078	0.0002	0.0061	0.0001	0.0013	0.0004	0.0030	0.0003	0.0055	0.0002	0.0053	0.0011
38.90	0.04	0.0070	0.0005	0.0078	0.0002	0.0063	0.0001	0.0015	0.0002	0.0047	0.0003	0.0047	0.0002	0.0053	0.0009
40.32	0.05	0.0078	0.0003	0.0078	0.0002	0.0060	0.0001	0.0016	0.0001	0.0042	0.0004	0.0050	0.0002	0.0054	0.0010
43.2	0.1	0.0085	0.0003	0.0081	0.0002	0.0062	0.0001	0.0024	0.0002	0.0058	0.0003	0.0050	0.0001	0.0060	0.0009
44.81	0.07	0.0080	0.0003	0.0085	0.0002	0.0061	0.0001	0.0034	0.0002	0.0058	0.0002	0.0043	0.0001	0.0060	0.0008
47.40	0.07	0.0072	0.0004	0.0086	0.0001	0.0055	0.0001	0.0046	0.0003	0.0060	0.0003	0.0052	0.0003	0.0062	0.0006
47.90	0.12	0.0068	0.0004	0.0080	0.0001	0.0057	0.0001	0.0036	0.0002	0.0057	0.0002	0.0044	0.0001	0.0057	0.0007
49.02	0.07	0.0058	0.0003	0.0082	0.0001	0.0058	0.0001	0.0037	0.0002	0.0064	0.0002	0.0040	0.0002	0.0057	0.0007
52.8	0.1	0.0057	0.0002	0.0097	0.0003	0.0054	0.0001	0.0037	0.0006	0.0068	0.0001	0.0041	0.0001	0.0059	0.0009
55.24	0.08	0.0064	0.0003	0.0094	0.0002	0.0054	0.0001	0.0033	0.0004	0.0072	0.0003	0.0042	0.0002	0.0060	0.0009
60.14	0.02	0.0066	0.0003	0.0092	0.0002	0.0052	0.0001	0.0040	0.0003	0.0080	0.0002	0.0040	0.0002	0.0062	0.0009
66.00	0.08	0.0067	0.0003	0.0086	0.0002	0.0048	0.0001	0.0046	0.0003	0.0070	0.0003	0.0040	0.0002	0.0060	0.0007

67.5	0.1	0.0056	0.0005	0.0085	0.0002	0.0048	0.0001	0.0044	0.0004	0.0071	0.0002	0.0042	0.0002	0.0058	0.0007
69.27	0.08	0.0057	0.0003	0.0085	0.0001	0.0053	0.0001	0.0038	0.0004	0.0076	0.0002	0.0037	0.0002	0.0058	0.0008
71.1	0.1	0.0057	0.0003	0.0083	0.0001	0.0052	0.0001	0.0041	0.0002	0.0074	0.0002	0.0033	0.0002	0.0057	0.0008
73.86	0.08	0.0046	0.0004	0.0078	0.0001	0.0051	0.0001	0.0043	0.0003	0.0068	0.0005	0.0051	0.0001	0.0056	0.0006
76.46	0.01	0.0047	0.0004	0.0081	0.0001	0.0052	0.0001	0.0051	0.0002	0.007	0.001	0.0040	0.0003	0.0057	0.0007
77.78	0.01	0.0053	0.0003	0.0081	0.0001	0.0048	0.0001	0.0048	0.0003	0.0077	0.0005	0.0048	0.0005	0.0059	0.0006
78.6	0.1	0.0053	0.0004	0.0078	0.0001	0.0050	0.0001	0.0047	0.0003	0.0064	0.0005	0.0046	0.0004	0.0056	0.0005
81.33	0.01	0.0052	0.0002	0.0074	0.0001	0.0045	0.0001	0.0045	0.0003	0.0071	0.0004	0.0042	0.0006	0.0055	0.0006
83.51	0.00	0.0062	0.0002	0.0075	0.0001	0.0050	0.0001	0.0044	0.0003	0.0070	0.0002	0.0043	0.0004	0.0057	0.0006
85.56	0.01	0.0057	0.0003	0.0074	0.0001	0.0050	0.0001	0.0045	0.0003	0.0063	0.0002	0.0044	0.0003	0.0056	0.0005
88.60	0.17	0.0061	0.0002	0.0072	0.0001	0.0047	0.0001	0.0041	0.0002	0.0070	0.0002	0.0031	0.0002	0.0054	0.0007
90.63	0.11	0.0055	0.0002	0.0071	0.0001	0.0047	0.0001	0.0043	0.0003	0.0078	0.0003	0.0043	0.0003	0.0056	0.0006
92.64	0.13	0.0053	0.0002	0.0071	0.0001	0.0046	0.0001	0.0040	0.0002	0.0067	0.0001	0.0034	0.0001	0.0052	0.0006
95.11	0.16	0.0064	0.0001	0.0073	0.0001	0.0050	0.0002	0.0042	0.0003	0.0072	0.0002	0.0035	0.0002	0.0056	0.0007
98.10	0.02	0.0055	0.0002	0.0074	0.0001	0.0050	0.0002	0.0040	0.0003	0.0066	0.0002	0.0045	0.0002	0.0055	0.0005
100.14	0.13	0.0060	0.0001	0.0072	0.0001	0.0047	0.0001	0.0040	0.0002	0.0067	0.0002	0.0036	0.0001	0.0054	0.0006

Supplementary Table 3. Temperature (T) evolution of the lattice strain parameters $Q(hkl)$ of hcp-Fe-2Si-0.4C at 36±3 GPa.

T, K	$Q(100)$	$\Delta Q(100)$	$Q(002)$	$\Delta Q(100)$	$Q(101)$	$\Delta Q(101)$	$Q(102)$	$\Delta Q(102)$	$Q(110)$	$\Delta Q(110)$	$Q(103)$	$\Delta Q(103)$	$Q(112)$	$\Delta Q(112)$
373	0.0055	0.0002	0.0064	0.0005	0.0053	0.0001	0.0046	0.0001	0.0048	0.0002	0.0056	0.0001	0.0065	0.0002

473	0.0050	0.0002	0.0057	0.0003	0.0045	0.0001	0.0044	0.0002	0.0054	0.0002	0.0051	0.0002	0.0066	0.0002
573	0.0055	0.0003	0.0057	0.0001	0.0052	0.0001	0.0041	0.0002	0.0053	0.0002	0.0056	0.0002	0.0063	0.0003
773	0.0057	0.0002	0.0054	0.0001	0.0045	0.0001	0.0038	0.0002	0.0047	0.0002	0.0048	0.0002	0.0055	0.0003
873	0.0055	0.0002	0.0054	0.0002	0.0042	0.0001	0.0033	0.0002	0.0047	0.0002	0.0050	0.0002	0.0052	0.0002
973	0.0051	0.0004	0.0050	0.0003	0.0043	0.0001	0.0045	0.0002	0.0053	0.0002	0.0051	0.0002	0.0051	0.0003
1073	0.0050	0.0002	0.0040	0.0002	0.0047	0.0001	0.0050	0.0002	0.0051	0.0002	0.0050	0.0002	0.0055	0.0002

Supplementary Table 4. Stiffness tensor components (C_{ij}) of hcp-Fe-2Si-0.4C at given pressures (P) and temperatures (T). The calculation is based on refs [1–3].

P, GPa	T, K	C_{11}, GPa	ΔC_{11}, GPa	C_{12}, GPa	ΔC_{12}, GPa	C_{44}, GPa	ΔC_{44}, GPa	C_{13}, GPa	ΔC_{13}, GPa	C_{33}, GPa	ΔC_{33}, GPa
18±3	300	655	13	243	14	129	6	210	7	710	30
41±4	300	785	15	321	15	157	7	274	8	850	30
49.4±0.5	300	830	16	348	15	166	7	297	8	910	30
62±6	300	899	18	389	16	181	8	331	9	980	30
80±8	300	1000	20	450	17	202	9	381	9	1100	30
87±9	300	1030	20	470	17	209	9	398	10	1130	30
91±9	300	1060	20	486	17	215	9	411	10	1160	30
98±10	300	1090	20	506	18	221	10	428	10	1200	30
98±10	300	1100	20	508	18	222	10	430	10	1210	30
105±11	300	1130	30	531	19	230	10	449	11	1250	30
107±11	300	1140	30	536	19	232	10	452	11	1260	30
108±11	300	1150	30	540	19	233	10	456	11	1260	30

115±12	300	1190	30	563	19	241	11	475	11	1310	30
128±13	300	1250	30	600	20	255	12	509	12	1380	30
36.33±0.05	1100	702	20	330	20	113	8	280	15	760	40
37.28±0.05	1100	707	20	330	20	114	8	282	15	766	40
38.90±0.04	1100	716	20	340	20	116	8	286	15	776	40
40.32±0.04	1100	724	20	340	20	118	8	290	15	785	40
43.2±0.1	1100	740	20	350	20	121	8	298	15	803	40
44.81±0.07	1100	750	20	360	20	123	8	303	15	813	40
47.40±0.07	1100	764	20	360	20	126	8	310	15	830	40
47.90±0.12	1100	770	20	370	20	127	8	311	15	833	40
49.02±0.07	1100	770	20	370	20	128	8	314	15	840	40
52.8±0.1	1100	790	20	380	30	133	8	325	15	864	40
55.24±0.08	1100	810	20	390	30	136	10	331	16	880	40
60.14±0.02	1100	830	20	410	30	141	10	345	16	910	40
66.00±0.08	1100	870	20	430	30	148	10	361	16	940	40
67.5±0.1	1100	870	20	430	30	150	10	365	16	950	40
69.27±0.08	1100	880	20	440	30	152	10	370	16	970	40
71.1±0.01	1100	890	20	440	30	154	10	375	16	980	40
73.86±0.08	1100	910	20	450	30	157	10	382	16	990	40
76.46±0.01	1100	920	20	460	30	160	10	390	16	1010	40
77.78±0.01	1100	930	30	460	30	162	10	393	16	1020	40
78.6±0.1	1100	940	30	470	30	163	10	395	16	1020	40
81.33±0.01	1100	950	30	480	30	166	10	402	16	1040	40
83.51±0.01	1100	960	30	480	30	168	10	408	16	1050	40
85.56±0.01	1100	970	30	490	30	171	10	414	16	1060	40
88.60±0.17	1100	990	30	500	30	174	10	422	16	1080	40
90.63±0.11	1100	1000	30	510	30	176	10	428	16	1100	40
92.64±013	1100	1010	30	510	30	180	10	433	17	1110	40
95.11±0.16	1100	1020	30	520	30	181	11	440	17	1120	40
98.10±0.02	1100	1040	30	530	30	185	11	448	17	1140	40
100.14±0.13	1100	1050	30	540	30	187	11	453	16	1150	40

Supplementary Table 5. Stiffness tensor components (C_{ij}) of hcp-Fe-2Si-0.4C at given temperatures and 329/364 GPa. The calculation is based on refs [1–3].

Pressure, GPa / Temperature, K	C_{11}, GPa	ΔC_{11}, GPa	C_{12}, GPa	ΔC_{12}, GPa	C_{13}, GPa	ΔC_{13}, GPa	C_{33}, GPa	ΔC_{33}, GPa	C_{44}, GPa	ΔC_{44}, GPa
329 / 3000	2010	90	1290	50	1089	40	2240	80	320	40
329 / 4000	1920	90	1310	50	1110	40	2150	70	280	40
329 / 5000	1815	90	1330	40	1120	40	2050	60	230	30
329 / 5500	1760	90	1340	50	1130	40	2000	70	200	30
364 / 3000	2150	30	1380	30	1170	20	2390	50	348	9
364 / 4000	2050	30	1400	20	1184	18	2300	30	301	9
364 / 5000	1950	20	1422	7	1198	17	2200	20	253	7
364 / 5500	1895	19	1432	9	1210	30	2150	40	230	6

Supplementary Table 6. Calculation of the yield strength (t) of hcp-Fe-2Si-0.4C at indicated temperatures and 329/364 GPa by Eq. (4). The calculation of the shear moduli, G_H, is based on the stiffness tensor component from **Supplementary Table 5**. $\langle Q(hkl)\rangle$ is the averaged lattice strain parameter at corresponding conditions.

Pressure, GPa / Temperature, K	G_H, GPa	ΔG_H, GPa	$\langle Q(hkl)\rangle$	$\Delta\langle Q(hkl)\rangle$	t, GPa	Δt, GPa
329 / 3000	380	20	0.0049	0.0005	11.2	1.3
329 / 4000	330	20	0.0040	0.0007	7.9	1.4
329 / 5000	270	20	0.0032	0.0009	5.2	1.5
329 / 5500	240	20	0.0027	0.0010	4.0	1.5
364 / 3000	406	8	0.0049	0.0005	12.0	1.2
364 / 4000	352	6	0.0041	0.0007	8.6	1.5
364 / 5000	296	4	0.0032	0.0009	5.7	1.6
364 / 5500	267	5	0.0028	0.0010	4.4	1.6

Supplementary Table 7. Sound velocities anisotropy (V_P, V_{S1}, V_{S2}) and maximum shear wave splitting ($V_{S1} - V_{S2}$) in hcp-Fe-2Si-0.4C polycrystalline aggregate at given pressures (P) and 300 K.

P, GPa	V_P anisotropy, %	V_{S1} anisotropy, %	V_{S2} anisotropy, %	Maximum shear wave splitting, $V_{S1} - V_{S2}$, km/s
18±3	0.52±0.05	1.36±0.13	0.61±0.06	0.046±0.004
41±4	0.66±0.05	1.81±0.14	0.86±0.07	0.063±0.004
49.4±0.5	0.26±0.05	0.71±0.11	0.26±0.05	0.027±0.003
62±6	0.22±0.05	0.62±0.11	0.21±0.07	0.025±0.003
80±8	0.27±0.03	0.40±0.06	0.72±0.07	0.026±0.002
87±9	0.06±0.03	0.08±0.06	0.07±0.06	0.006±0.003
91±9	0.31±0.04	0.44±0.09	0.78±0.06	0.0028±0.003
98±10	0.21±0.04	0.25±0.11	0.44±0.09	0.015±0.006
98±10	0.17±0.04	0.15±0.12	0.23±0.11	0.010±0.008
105±11	0.40±0.03	0.45±0.12	0.94±0.08	0.036±0.001
107±11	0.16±0.03	0.10±0.09	0.24±0.02	0.009±0.004
108±11	0.14±0.02	0.38±0.02	0.21±0.12	0.014±0.005
115±12	0.12±0.02	0.23±0.08	0.16±0.08	0.010±0.006
128±13	0.61±0.01	0.54±0.18	1.40±0.07	0.059±0.002

Supplementary Table 8. Sound velocities anisotropy (V_P, V_{S1}, V_{S2}) and maximum shear wave splitting ($V_{S1} - V_{S2}$) in hcp-Fe-2Si-0.4C polycrystalline aggregate at given pressures and 1100 K.

P, GPa	V_P anisotropy, %	V_{S1} anisotropy, %	V_{S2} anisotropy, %	Maximum shear wave splitting, $V_{S1} - V_{S2}$, km/s
36.33±0.05	1.13±0.13	4.4±0.3	3.23±0.14	0.182±0.001
37.28±0.05	0.98±0.12	3.8±0.2	2.82±0.14	0.160±0.002
38.90±0.04	0.9±0.2	3.93±0.06	2.85±0.13	0.166±0.004
40.32±0.05	0.93±0.16	3.86±0.09	2.86±0.06	0.164±0.002
43.2±0.1	1.12±0.10	4.1±0.3	2.93±0.10	0.163±0.002
44.81±0.07	1.08±0.10	4.0±0.3	2.83±0.09	0.158±0.003
47.40±0.07	1.02±0.10	3.7±0.3	2.66±0.07	0.150±0.002
47.90±0.12	0.88±0.14	3.50±0.07	2.60 ±0.02	0.151±0.005
49.02±0.07	1.04±0.11	3.8±0.2	2.69±0.05	0.151±0.003
52.8±0.1	1.11±0.07	3.9±0.3	2.53±0.11	0.140±0.001
55.24±0.08	1.03±0.07	3.6±0.2	2.41±0.07	0.135±0.002
60.14±0.02	1.0±0.1	3.4±0.2	2.31±0.02	0.131±0.005
66.00±0.08	0.92±0.07	3.1±0.2	1.98±0.05	0.111±0.003
67.5±0.1	0.87±0.07	2.9±0.2	1.87±0.01	0.106±0.005
69.27±0.08	0.96±0.08	3.2±0.2	2.06±0.01	0.117±0.007
71.1±0.1	0.92±0.07	3.0±0.2	1.93±0.01	0.109±0.006
73.86±0.08	0.68±0.07	2.2±0.2	1.45±0.04	0.084±0.007
76.46±0.01	0.95±0.04	3.0±0.2	1.81±0.02	0.101±0.004
77.78±0.01	1.02±0.06	3.2±0.2	1.95±0.02	0.109±0.007
78.6±0.1	0.87±0.04	2.8±0.2	1.61±0.02	0.091±0.005
81.33±0.01	0.99±0.04	3.1±0.2	1.79±0.05	0.100±0.007
83.51±0.01	0.93±0.04	2.9±0.2	1.64±0.04	0.092±0.008
85.56±0.01	0.90±0.03	2.8±0.2	1.56±0.01	0.089±0.009
88.60±0.17	0.78±0.02	2.4±0.2	1.32±0.05	0.077±0.009

90.63±0.11	0.94±0.02	2.9±0.2	1.57±0.05	0.094±0.009
92.64±0.13	0.92±0.04	2.7±0.2	1.51±0.07	0.091±0.009
95.11±0.16	0.96±0.03	2.9±0.2	1.53±0.10	0.096±0.010
98.10±0.02	0.88±0.03	2.6±0.2	1.36±0.07	0.089±0.010
100.14±0.13	0.76±0.11	2.4±0.1	1.4±0.4	0.084±0.009

Supplementary Table 9. Experimental pressure-temperature conditions (P, T) together with the unit cell volume (V), averaged lattice strain parameter $\langle Q(hkl)\rangle$, Voigt-Reuss-Hill shear modulus G_H, and yield strength t of the hcp-Fe-2Si-0.4C alloy. Note, that here $\langle Q(hkl)\rangle$ is calculated over (100), (002) and (103) lattice planes.

P, GPa	ΔP, GPa	T, K	V, Å^3	ΔV, Å^3	$\langle Q(hkl)\rangle$	$\Delta\langle Q(hkl)\rangle$	G_H, GPa	ΔG_H, GPa	t, GPa	Δt, GPa
18	3	300	20.967	0.011	0.0070	0.0012	177	8	7.5	1.3
41	4	300	19.46	0.13	0.0070	0.0015	207	9	9	1.9
49.4	0.5	300	19.07	0.03	0.0072	0.0011	217	10	9.3	1.4
62	6	300	18.50	0.03	0.0070	0.0004	233	10	10.0	0.7
80	8	300	17.76	0.03	0.0083	0.0005	256	12	13.0	1.0
87	9	300	17.53	0.03	0.007	0.0011	263	12	12	1.8
91	9	300	17.36	0.016	0.010	0.0009	269	13	16	1.7
98	10	300	17.160	0.011	0.0084	0.0014	277	13	14	2.4
98	10	300	17.14	0.02	0.0087	0.0011	277	13	14	1.9
105	11	300	16.92	0.05	0.009	0.0022	286	14	16	3.8
107	11	300	16.88	0.03	0.0086	0.0014	287	14	15	2.5
108	11	300	16.84	0.05	0.0079	0.0015	289	14	14	2.7
115	12	300	16.63	0.01	0.0075	0.0015	297	15	13	2.8
128	13	300	16.283	0.007	0.0080	0.0013	312	16	15	2.6
36.33	0.05	1117	20.014	0.002	0.0069	0.0009	161	13	6.7	1.0

37.28	0.05	1119	19.945	0.003	0.0071	0.0008	162	14	7.0	1.0
38.90	0.04	1117	19.870	0.003	0.007	0.0009	164	14	6.4	1.1
40.32	0.05	1117	19.701	0.002	0.007	0.0009	166	14	6.8	1.1
43.2	0.1	1114	19.574	0.004	0.0072	0.0011	170	14	7.3	1.3
44.81	0.07	1112	19.454	0.002	0.0070	0.0013	172	14	7.1	1.5
47.40	0.07	1110	19.361	0.002	0.007	0.0010	175	14	7.4	1.2
47.90	0.12	1108	19.264	0.002	0.0064	0.0011	176	14	6.7	1.2
49.02	0.07	1110	19.241	0.002	0.0060	0.0012	177	14	6.4	1.4
52.8	0.1	1103	18.967	0.002	0.0065	0.0017	182	14	7	1.9
55.24	0.08	1100	18.924	0.001	0.0067	0.0015	185	14	7.4	1.8
60.14	0.02	1098	18.688	0.002	0.0066	0.0015	191	14	7.6	1.8
66.00	0.08	1097	18.505	0.002	0.0064	0.0013	200	15	7.6	1.7
67.5	0.1	1097	18.447	0.002	0.0061	0.0013	201	15	7.3	1.6
69.27	0.08	1097	18.357	0.001	0.0060	0.0014	203	15	7.2	1.8
71.1	0.1	1097	18.270	0.001	0.0058	0.0014	205	15	7.1	1.9
73.86	0.08	1098	18.212	0.001	0.006	0.0010	210	15	7.3	1.4
76.46	0.01	1097	18.118	0.002	0.0056	0.0013	212	15	7.1	1.7
77.78	0.01	1096	18.014	0.005	0.0061	0.0010	213	15	7.8	1.4
78.6	0.1	1096	18.044	0.003	0.006	0.0010	214	15	7.6	1.4
81.33	0.01	1097	17.948	0.002	0.006	0.0010	218	16	7.3	1.4
83.51	0.00	1097	17.848	0.002	0.006	0.0009	220	16	8.0	1.4
85.56	0.01	1097	17.770	0.002	0.006	0.0009	223	16	7.8	1.3
88.60	0.17	1097	17.706	0.002	0.0055	0.0012	227	16	7.5	1.8
90.63	0.11	1095	17.606	0.002	0.0056	0.0008	230	16	7.7	1.3
92.64	0.13	1097	17.547	0.003	0.0053	0.0011	232	16	7.3	1.6
95.11	0.16	1096	17.445	0.002	0.0057	0.0011	235	16	8.1	1.7
98.10	0.02	1096	17.415	0.004	0.0058	0.0009	238	17	8.3	1.4
100.14	0.13	1096	17.351	0.003	0.0056	0.0011	241	17	8.1	1.6

Supplementary Table 10. Lattice parameter (a) of gold pressure standard together with the derived pressures (P)[4], experimental temperature (T) conditions and the unit-cell volume (V) of hcp-Fe-2Si-0.4C at 36±3 GPa.

a, Au (Å)	Δa, Au (Å)	P, GPa	ΔP, GPa	T, K	V, $Å^3$	ΔV, $Å^3$
3.9086	0.0004	33.42	0.11	373	20.186	0.002
3.9074	0.0003	33.70	0.08	473	19.700	0.002
3.8906	0.0003	38.2	0.1	573	19.570	0.002
3.8976	0.0005	36.30	0.14	773	19.556	0.003
3.8877	0.0003	39.0	0.1	873	19.521	0.002
3.8979	0.0004	36.2	0.1	973	19.506	0.003
3.8872	0.0004	39.16	0.12	1073	19.466	0.003

Supplementary Table 11. Lattice parameter (a) of gold pressure standard together with the derived pressures (P)[4] at ~1100 K between 36–100 GPa.

a, Au (Å)	Δa, Au (Å)	T, K	P, GPa	ΔP, GPa
3.8974	0.0002	1117	36.33	0.05
3.8940	0.0002	1119	37.28	0.05
3.8882	0.0001	1117	38.90	0.04
3.8831	0.0002	1117	40.32	0.05
3.8734	0.0003	1114	43.2	0.1
3.8680	0.0002	1112	44.81	0.07
3.8596	0.0002	1110	47.40	0.07
3.8580	0.0004	1108	47.90	0.12
3.8545	0.0002	1110	49.02	0.07
3.8429	0.0003	1103	52.8	0.1
3.8358	0.0002	1100	55.24	0.08
3.8220	0.0001	1098	60.14	0.02
3.8064	0.0002	1097	66.00	0.08
3.8027	0.0002	1097	67.5	0.1
3.7981	0.0002	1097	69.27	0.08
3.7936	0.0001	1097	71.1	0.1
3.7868	0.0001	1098	73.86	0.08
3.7806	0.0002	1097	76.46	0.01

3.7775	0.0001	1096	77.78	0.01
3.7756	0.0001	1096	78.6	0.1
3.7695	0.0001	1097	81.33	0.01
3.7646	0.0003	1097	83.51	0.00
3.7601	0.0003	1097	85.56	0.01
3.7536	0.0004	1097	88.60	0.17
3.7493	0.0002	1095	90.63	0.11
3.7451	0.0003	1097	92.64	0.13
3.7401	0.0003	1096	95.11	0.16
3.7342	0.0001	1096	98.10	0.02
3.7301	0.0003	1096	100.14	0.13

Supplementary Table 12. Parameters for the determination of the pressure dependence of stiffness tensor components (C_{ij}) of hcp-Fe-2Si-0.4C using the equation $C_{ij} = aP^2 + bP + c$, where c is temperature-dependent, P is pressure and a, b are parameters dependent on Si and C content. Temperature-dependent parameter c is determined from data of ref [2]. Parameters a, b were determined from refs [1,3].

Stiffness parameter	c (300 K)	Δc	c (1100 K)	Δc	b	Δb	a	Δa
C_{11}	551	12	496	18	5.8	0.20	-0.0020	0.0005
C_{12}	181	14	204	24	3.40	0.11	-0.0008	0.0002
C_{44}	106	6	69	7	1.24	0.08	-0.0006	0.0002
C_{13}	160	7	179	15	2.78	0.08	-0.0004	0.0002
C_{33}	591	27	528	40	6.48	0.14	-0.0024	0.0003

Supplementary Table 13. Parameters used for calculation of viscosity of hcp-Fe-2Si-0.4C at core conditions (Eq. (6)–(12), **Fig. 7**).

	Value	Refs and notes
M, molar mass, kg/mol	0.055	this study
V_0, unit cell volume of the non-compressed state, Å^3	22.56±0.17	[5]
K_0', bulk modulus pressure derivative	3.7±0.3	[5]
K_0, bulk modulus, GPa	215±18	[5]
θ_0, Debye temperature at ambient pressure, K	417	[6]
γ_0, Grüneisen parameter at 1 bar	2.0±0.1	[7]
q, scaling parameter	1.0±0.2	[7]
ρ, density, g/cm^3	13.89±0.16	[5–7]
V_D, Debye sound velocity, km/s	5.8±1.0	[5]
ν_D, Debye frequency, THz	19.2	[5]
τ_P, Peierls stress, GPa	4.4±1.6	this study
b, Burgers vector, Å	2.10±0.01	[5,8]
G_H, shear modulus, GPa	267±5	this study

ΔH_0, activation enthalpy, eV	4.9±0.1	[9]
a', Peierls barrier width, Å	2.10±0.01	[5,10]
L, dislocation length, μm	0.5	[11]

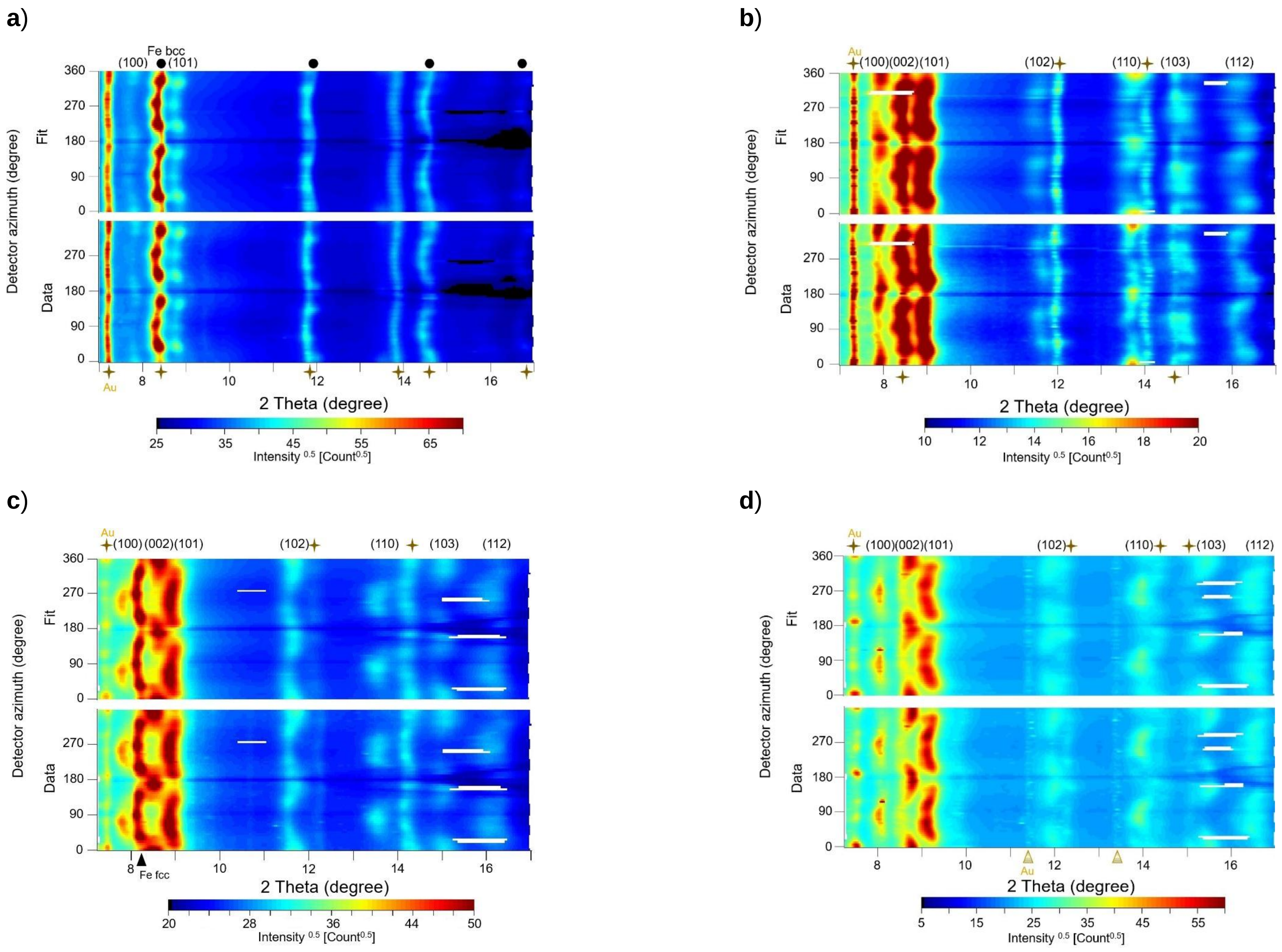
a)
Fe bcc
(100)
(101)
Detector azimuth (degree)
Fit
Data
360
270
180
90
0
Au
8
10
12
14
16
2 Theta (degree)
25
35
45
55
65
Intensity 0.5 [Count0.5]
b)
Au
(100)(002)(101)
(102)
(110)
(103)
(112)
Detector azimuth (degree)
Fit
Data
2 Theta (degree)
10
12
14
16
18
20
Intensity 0.5 [Count0.5]
c)
Au
(100)(002)(101)
(102)
(110)
(103)
(112)
Detector azimuth (degree)
Fit
Data
Fe fcc
2 Theta (degree)
20
28
36
44
50
Intensity 0.5 [Count0.5]
d)
Au
(100)(002)(101)
(102)
(110)
(103)
(112)
Detector azimuth (degree)
Fit
Data
Au
2 Theta (degree)
5
15
25
35
45
55
Intensity 0.5 [Count0.5]

Supplementary Fig. 1 | Unrolled diffraction patterns (bottom) together with the best-fit models (top) of Fe-2Si-0.4C upon compression and heating. Numbers in brackets (e.g., 110) indicate the Laue indices of the Bragg reflections of the compressed hcp-Fe-2Si-0.4C sample. White horizontal lines mask reflections from diamonds **a** Onset of the transformation of bcc-Fe-2Si-0.4C to hcp structure. Pattern collected at 300 K and 11 GPa, black circles indicate diffraction lines from bcc-Fe-2Si-0.4C sample (golden crosses – Au pressure standard reflections). **b** Completion of bcc transformation to hcp-Fe-2Si-0.4C. Pattern collected at 300 K and 27 GPa (golden crosses – Au pressure standard reflections). **c** Appearance of fcc-Fe-2Si-0.4C upon heating. Pattern collected at 1013 K and 39 GPa, black triangles indicate diffraction lines from fcc-Fe-2Si-0.4C sample (golden crosses – Au pressure standard reflections). **d** Completion of fcc-Fe-2Si-0.4C transformation back to hcp structure. Pattern collected at 1100 K and 55 GPa (golden crosses – Au pressure standard reflections). The golden pattern-filled triangles below indicate Au outside of the pressure chamber.

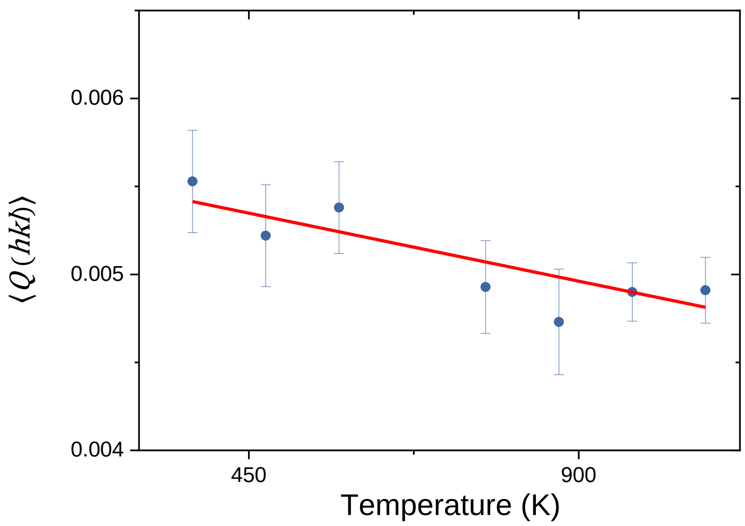

Supplementary Fig. 2 | Temperature evolution of hcp-Fe-2Si-0.4C averaged lattice strain parameter $\langle Q(hkl) \rangle$ at 36±3 GPa. The red line is the linear fit of the data.

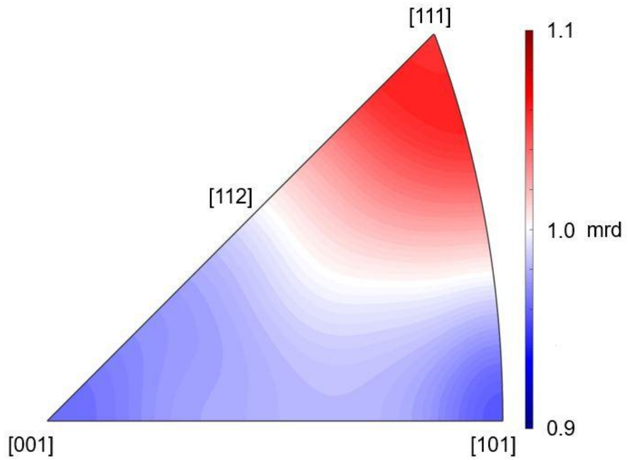

Supplementary Fig. 3 | Lattice-preferred orientation in bcc Fe-2Si-0.4C before compression. Inverse pole figure of the compression direction at 300 K and ambient pressure, 10^{-4} GPa. Note, that the texture intensity is below 1.1 mrd.

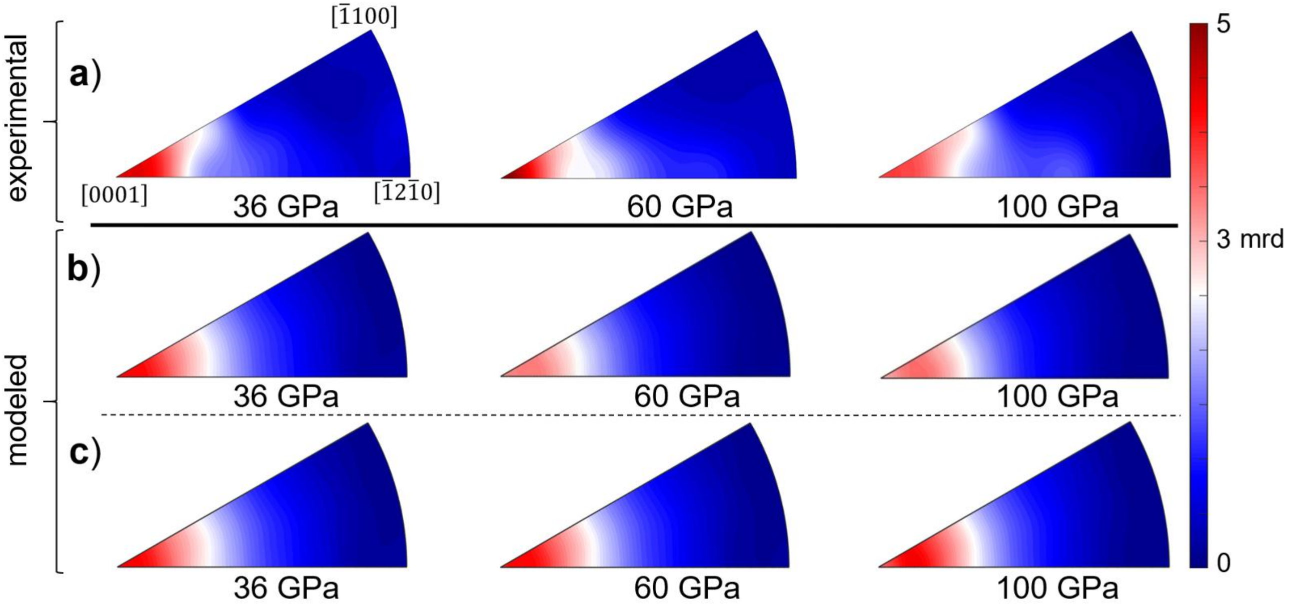

Supplementary Fig. 4 | Lattice-preferred orientation in hcp-Fe-2Si-0.4C alloy at high pressures and 1100 K. Inverse pole figures of the compression direction showing experimental deformation textures in hcp-Fe-2Si-0.4C (**a**), the deformation textures resulting from the EVPSC modeling considering basal and addition of prismatic and pyramidal slips (**b**), or considering basal and addition of prismatic slip and compressive twinning (**c**) at indicated pressures. The texture is expressed in multiples of a random distribution (mrd).

a)

b)

c)

d)

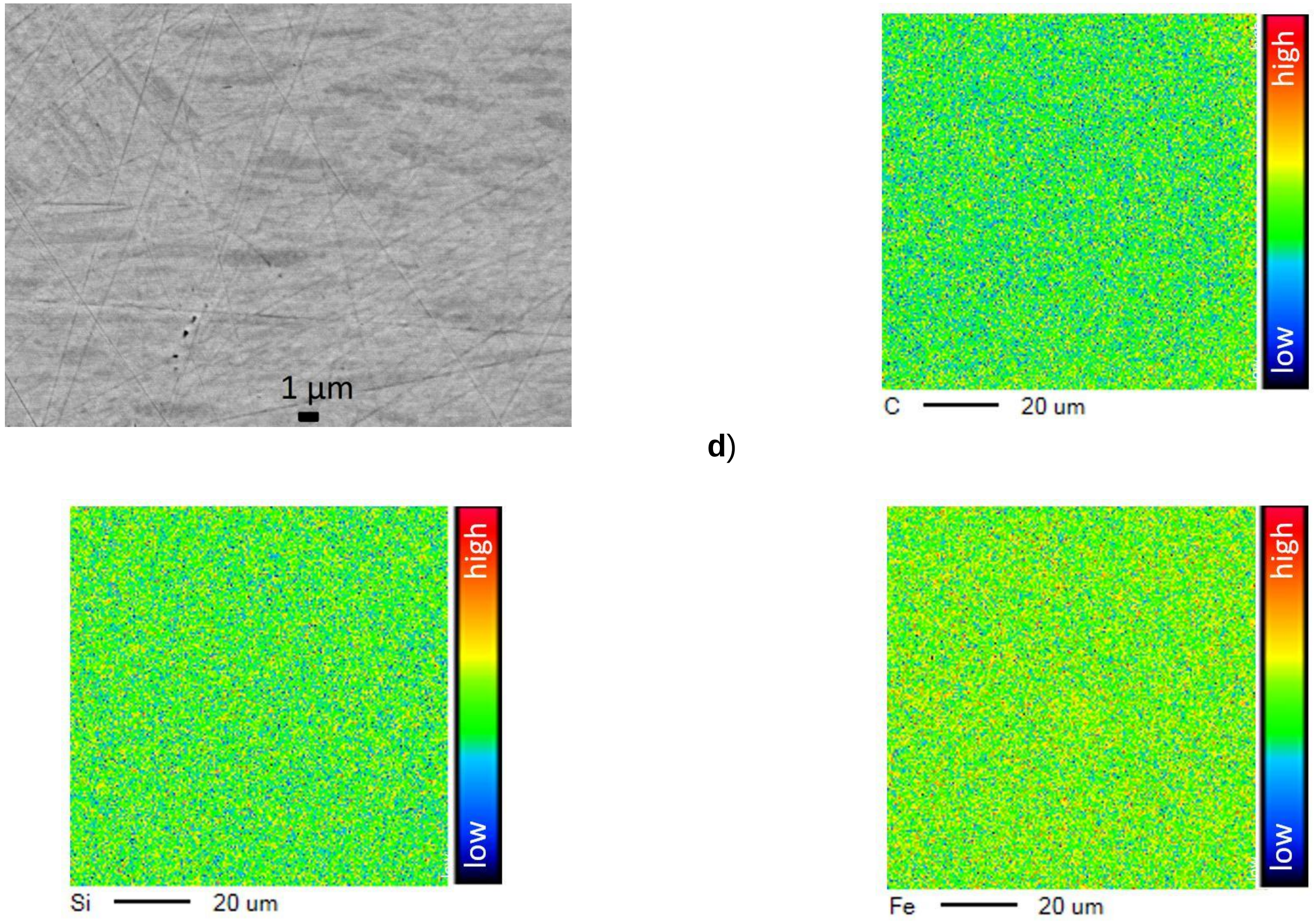

Supplementary Fig. 5 | SEM and the electron microprobe compositional analysis. **a** Backscattered electron image of the Fe-2Si-0.4C sample after the synthesis. (**b**), (**c**), and (**d**) are compositional qualitative X-ray distribution maps for C, Si and Fe, respectively.

Scales on the right in (**b**), (**c**), and (**d**) correspond to relative element concentration: low (blue) or high (red). The center of all four images is the same.

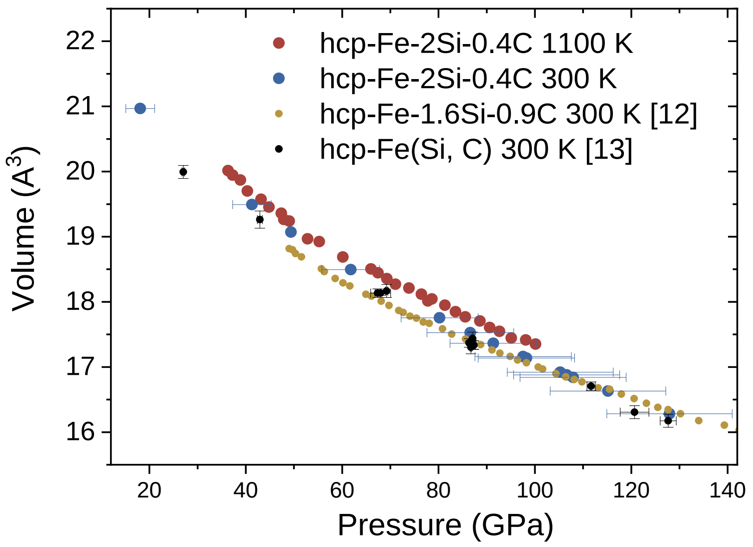

Supplementary Fig. 6 | The pressure dependence of the unit-cell volume of hcp-Fe-2Si-0.4C, hcp-Fe-1.6Si-0.9C[12] and hcp-Fe(Si, C)[13] at indicated temperatures. The error bars are smaller than the symbols unless shown.

a)

b)

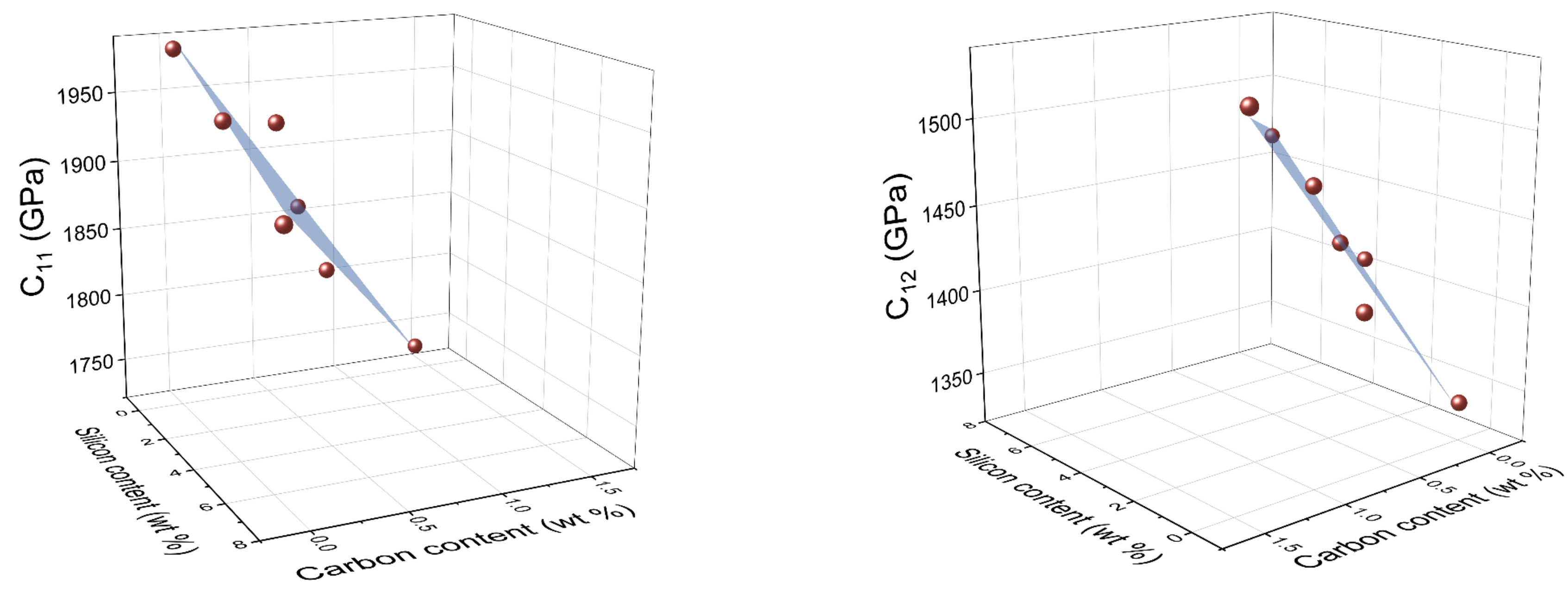
C11 (GPa)
1950
1900
1850
1800
1750
Silicon content (wt %)
Carbon content (wt %)
C12 (GPa)
1500
1450
1400
1350

c)

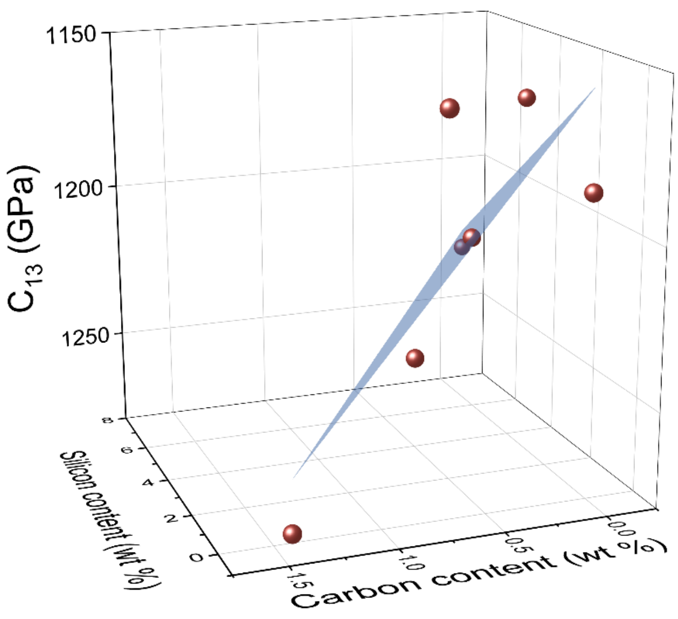
C13 (GPa)
1150
1200
1250
Silicon content (wt %)
0
2
4
6
8
Carbon content (wt %)
1.5
1.0
0.5
0.0

d)

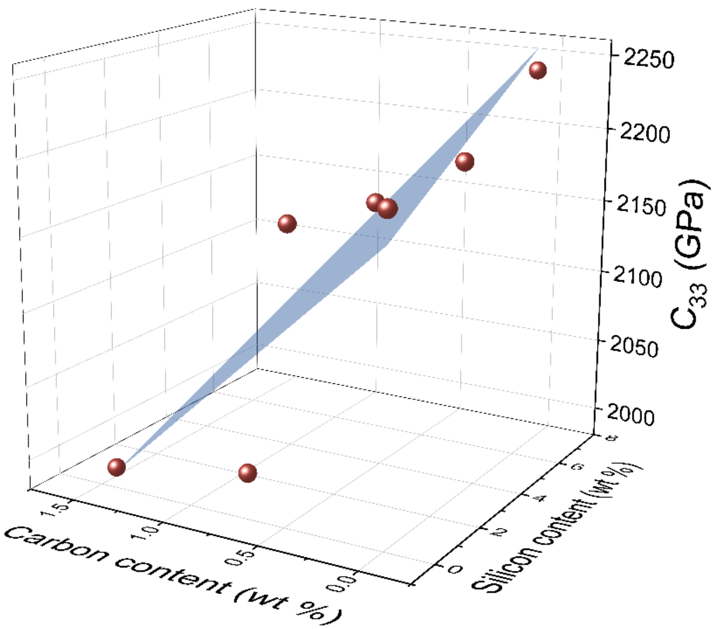
C33 (GPa)
2250
2200
2150
2100
2050
2000
Carbon content (wt %)
1.5
1.0
0.5
0.0
Silicon content (wt %)
0
2
4
6
8

e)

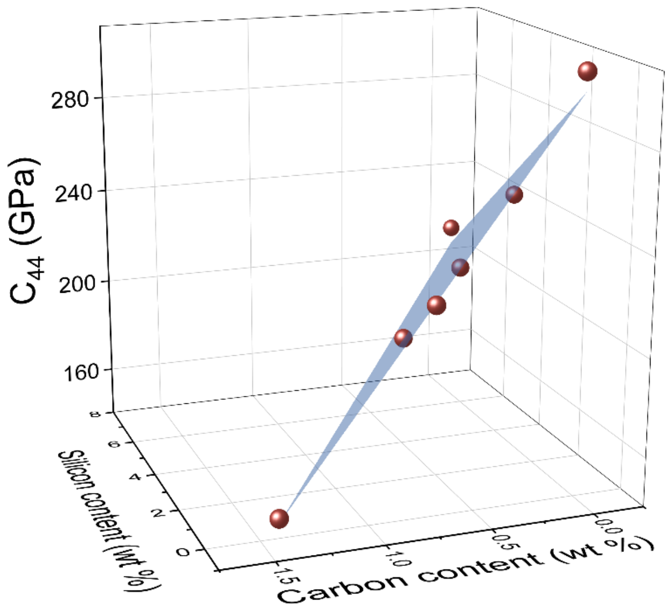

Supplementary Fig. 7 | Compositional dependence of hcp-Fe-Si-C alloys stiffness tensor components. Stiffness tensor components C_{11} (**a**), C_{12} (**b**), C_{13} (**c**), C_{33} (**d**) and C_{44} (**e**) (brown spheres) of iron alloys with carbon and silicon at 5500 K and 360 GPa, inferred from data of ref [2]. The blue planes represent the linear fit of C_{ij} dependence on silicon and carbon contents.

References

1. Caracas, R. The influence of carbon on the seismic properties of solid iron. *Geophysical Research Letters* **44**, 128–134 (2017).
2. Li, Y., Vočadlo, L. & Brodholt, J. P. The elastic properties of hcp-Fe alloys under the conditions of the Earth's inner core. *Earth and Planetary Science Letters* **493**, 118–127 (2018).
3. Tsuchiya, T. & Fujibuchi, M. Effects of Si on the elastic property of Fe at Earth's inner core pressures: First principles study. *Physics of the Earth and Planetary Interiors* **174**, 212–219 (2009).
4. Fei, Y. *et al.* Toward an internally consistent pressure scale. *Proceedings of the National Academy of Sciences* **104**, 9182–9186 (2007).
5. Müller, S. Equation of state and phonon Density of States of Fe-2wt%Si-0.4wt%C. Zenodo https://doi.org/10.5281/zenodo.15119916 (2025).
6. Dewaele, A. *et al.* Quasihydrostatic Equation of State of Iron above 2 Mbar. **215504**, 29–32 (2006).
7. Murphy, C. A., Jackson, J. M., Sturhahn, W. & Chen, B. Grüneisen parameter of hcp-Fe to 171 GPa. *Geophys. Res. Lett.* **38**, L24306 (2011).
8. Park, Y. *et al.* Viscosity of Earth's inner core constrained by Fe–Ni interdiffusion in Fe–Si alloy in an internal-resistive-heated diamond anvil cell. *American Mineralogist* **108**, 1064–1071 (2023).
9. Koizumi, H., Kirchner, H. O. K. & Suzuki, T. Kink pair nucleation and critical shear stress. *Acta Metallurgica et Materialia* **41**, 3483–3493 (1993).
10. Karato, S. *Deformation of Earth Materials: An Introduction to the Rheology of Solid Earth*. (Cambridge University Press, 2008).
11. Gleason, A. E. & Mao, W. L. Strength of iron at core pressures and evidence for a weak Earth's inner core. *Nature Geoscience* **6**, 571–574 (2013).

12. Pamato, M. G. *et al.* Equation of State of hcp Fe-C-Si Alloys and the Effect of C Incorporation Mechanism on the Density of hcp Fe Alloys at 300 K. *Journal of Geophysical Research: Solid Earth* **125**, 0–2 (2020).

13. Miozzi, F. *et al.* Eutectic melting of Fe-3 at% Si-4 at% C up to 200 GPa and implications for the Earth's core. *Earth and Planetary Science Letters* **544**, 116382 (2020).
